# Staged angioplasty in 2 patients with severe carotid artery stenosis: A case report and literature review

**DOI:** 10.1097/MD.0000000000040032

**Published:** 2024-10-04

**Authors:** Yong-Liang Zhou, Shi-Min Liu, Wen-Feng Cao, Xian-Min Cao, Ling-Feng Wu, An Wen

**Affiliations:** a Department of Neurology, Jiangxi Provincial People’s Hospital (The First Affiliated Hospital of Nanchang Medical College), Nanchang, People’s Republic of China; b Department of Neurology, Xiangya Hospital, Central South University, Jiangxi Hospital, National Regional Center for Neurological Diseases, Nanchang, People’s Republic of China; c Medical College of Nanchang University, Nanchang, People’s Republic of China.

**Keywords:** carotid artery stenosis, carotid artery stenting, hyperperfusion, staged angioplasty

## Abstract

**Rationale:**

Cerebral infarction is a common ischemic cerebrovascular disease, associated with high rates of morbidity, disability, and recurrence, that can seriously affect patient physical and mental health, as well as quality of life. Carotid artery stenosis is an independent risk factor of cerebral infarction. Following rapid developments in interventional technology and materials science, carotid artery stenting has arisen an important treatment option for carotid artery stenosis. However, surgery is associated with complications, such as postoperative hyperperfusion syndrome, which poses a serious threat to the life and health of patients. Staged angioplasty (SAP), that is, one-time revascularization of the carotid artery stenting, is divided into 2 stages. This method reduces the occurrence of hyperperfusion syndrome after stenting by increasing the ipsilateral cerebral blood flow in stages and gradually increasing the cerebral perfusion pressure.

**Patient concerns:**

Herein, we present 2 cases of elderly patients with severe carotid artery stenosis who underwent SAP to prevent hyperperfusion syndrome.

**Diagnoses:**

The final diagnosis was based on cervical vascular color Doppler ultrasonography, cervical vascular magnetic resonance angiography, and cerebral vascular digital subtraction angiography.

**Intervention:**

Both patients with severe carotid artery stenosis underwent a staged intravascular intervention.

**Outcomes:**

Both patients were followed up for 1 year, with neither developing any new cerebral infarction or recurrent stent restenosis.

**Lessons:**

When treating SAP, it is crucial to consider that patients with unstable carotid plaques may not be suitable for staging. Additionally, during phase II carotid stenting, it is important to assess any changes in the arterial morphology and select the appropriate device accordingly.

## 1. Introduction

Studies have indicated that 20% of the ischemic cerebrovascular diseases result from carotid artery stenosis.^[1,2]^ Carotid endarterectomy and carotid artery stenting (CAS) are both effective treatments for carotid artery stenoses.^[3]^ However, both are associated with a risk of postoperative hyperperfusion syndrome, a severe complication with an incidence ranging from 1.4% to 4.6%.^[4,5]^ Patients who develop this complication, particularly those with intracerebral hemorrhage as the primary manifestation, have a significantly elevated risk of mortality and disability.^[6]^

Staged angioplasty (SAP) for patients with severe carotid artery stenosis has been considered an effective strategy to eliminate the risk of hyperperfusion syndrome. SAP involves staged vascular dilatation, using a small balloon in the first phase, followed by CAS 2 to 4 weeks later.^[7]^

Since 2015, our hospital has implemented a staged treatment for patients with >90% stenosis, evident hypoperfusion in the responsible vascular supply area, advanced age, and slow intracranial blood flow. During this period, we performed 85 SAP and encountered a number of challenging cases, 12 of which were identified as difficult. This retrospective study focuses on the treatment process of 2 special cases with representative and teaching significance.

## 2. Case presentation

**Figure 1.**
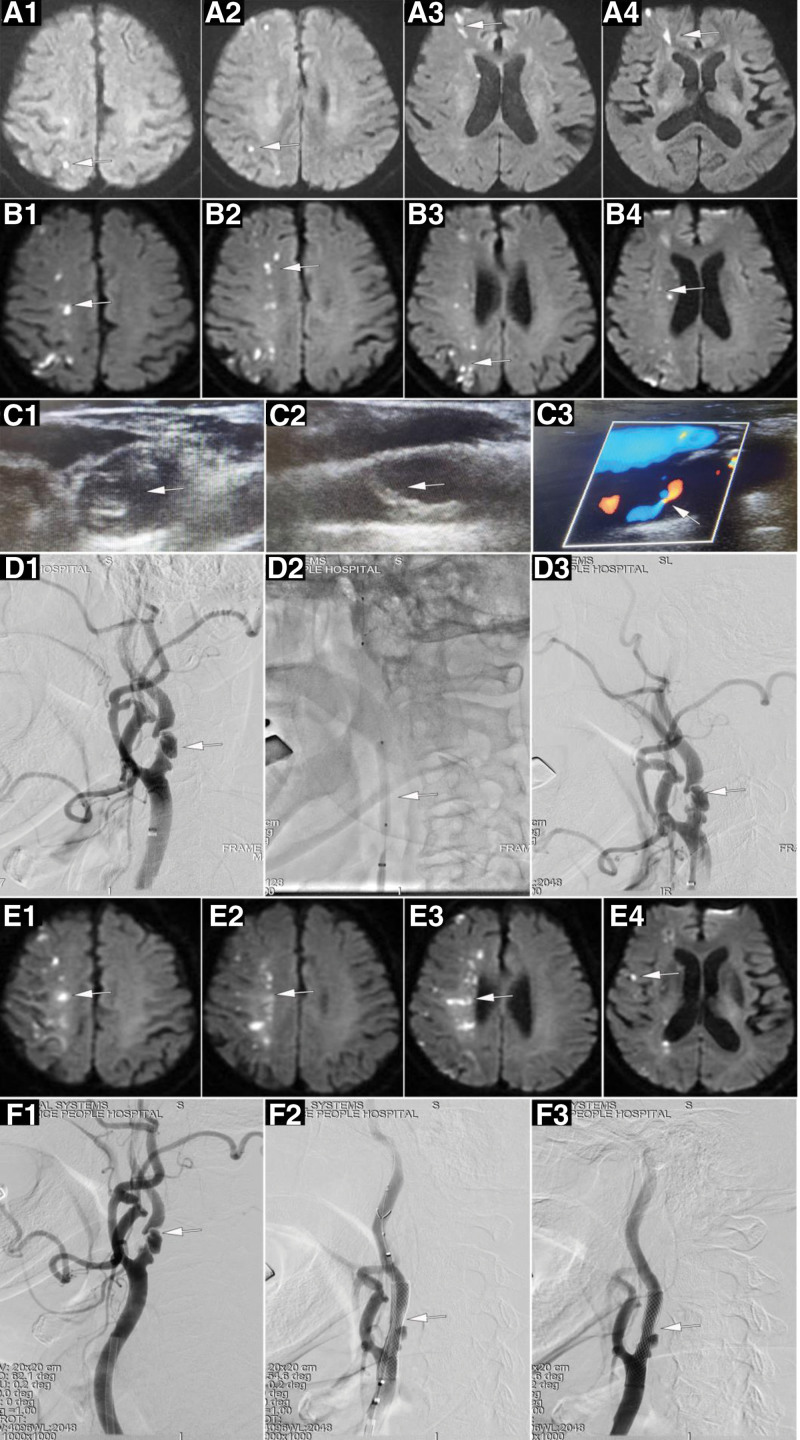
A 83-year-old man experienced a sudden onset of left-sided limb weakness of unknown origin. Diffusion-weighted image (DWI) showed Multiple spot-like infarcts in the right cerebral hemisphere (A1–A4). A follow-up DWI indicated more infarcts than before (B1–B4). Color Doppler ultrasound revealed severe stenosis of the right internal carotid artery (C1–C3). Left internal carotid artery was dilated by balloon (D1–D3). The patient experienced a recurrence of left-sided limb weakness, subsequent head MRI revealed a further enlargement of the infarction area (E1–E4). CAS was performed using WALLSTENT endoprosthesis (F1–F3).

The first case was an 83-year-old man who experienced sudden onset of left-sided limb weakness of unknown origin on April 15, 2022. Relevant symptoms included difficulty holding objects, unsteady gait, left-sided numbness, and drooling on one side of the mouth, which lasted for several minutes. He promptly sought medical attention at a local hospital, where magnetic resonance imaging (MRI) of the head revealed multiple punctate infarcts in the right cerebral hemisphere (Fig. 1A1–A4). The patient was prescribed aspirin and clopidogrel to reduce platelet aggregation, and symptomatic treatment to improve circulation. However, he later experienced recurrent symptoms on 5 occasions. The patient was transferred to our hospital on May 19, 2022. Upon admission, a follow-up head MRI on the same day revealed decreased perfusion in the right hemisphere, and an increased number of infarcts compared with previous scans (Fig. 1B1–B4). The treatment plan included oral aspirin, continuous intravenous tirofiban infusion, and subcutaneous injection of low-molecular-weight heparin (2000 IU/q12h) for anticoagulation. Additionally, clopidogrel genotype testing was conducted, with the results indicating intermediate metabolism. On May 21st, bedside cervical vascular color Doppler ultrasonography revealed severe stenosis of the right internal carotid artery. The initial segment displayed a markedly hypoechoic plaque with an incomplete fibrous cap on the transverse section. Dynamic imaging revealed floating movement of the plaque, while longitudinal imaging revealed plaque accumulation in the lumen. Color Doppler ultrasound further demonstrated a residual lumen diameter of <1 mm (Fig. 1C1–C3). On May 22nd, the treatment regimen was adjusted to include dual antiplatelet therapy with aspirin and ticagrelor. However, on May 24th, the patient experienced 2 episodes of left-sided limb weakness lasting >10 min. Follow-up cerebral artery angiography performed on May 25th subsequently revealed severe stenosis of the right internal carotid artery, making identification of the lumen challenging (Fig. 1D1). Despite intensive antiplatelet therapy, the patient continued to experience recurrent left-sided limb weakness, and MRI revealed multiple infarcts.

Under these circumstances, we planned to perform SAP on the right internal carotid artery with balloon dilation in the first phase, followed by CAS 2 to 4 weeks later. At 10:40 on May 25th, the patient underwent left internal carotid artery balloon dilation using a 4 × 30 mm balloon for 2 inflations (Fig. 1D2). However, following the procedure, a significant alteration in the morphology of the stenotic segment of the right internal was observed (Fig. 1D3). The patient underwent clinical examination immediately following the procedure, and the findings were consistent with those of the preoperative assessment. At 15:30 on the same day, the patient experienced recurrence of left-sided limb weakness, with motor strength assessed as 3 in the left upper limb and 5 in the lower limb. The symptoms persisted and remained unrelieved. Repeat MRI performed on May 26th indicated an increased number of punctate infarcts in the right cerebral hemisphere. On May 27th, the patient’s symptoms worsened, with left-sided limb weakness and motor strength assessed as 1 in the left upper limb and 3 in the lower limb. Subsequent head MRI revealed further enlargement of the infarcted area (Fig. 1E1–E4). CAS was performed using a WALLSTENT endoprosthesis (Boston Scientific, Marlborough, MA) at 21:00 am on May 27th. The procedure did not involve balloon predilation (Fig. 1F1–F2), and stent deployment was satisfactory, with a residual stenosis of approximately 20% (Fig. 1F3). There were no clinical manifestations of hyperperfusion syndrome such as headache, vomiting and severe fluctuation of blood pressure in the perioperative period. The patient ultimately recovered well, and was discharged. Follow-up was performed in the outpatient department 3 months after surgery, while reexamination of the carotid artery ultrasound showed unobstructed blood flow in the stent and no lumen stenosis. The muscle strength of the patient’s left limb recovered to grade 5, and he was able to take care of himself in daily life.

**Figure 2.**
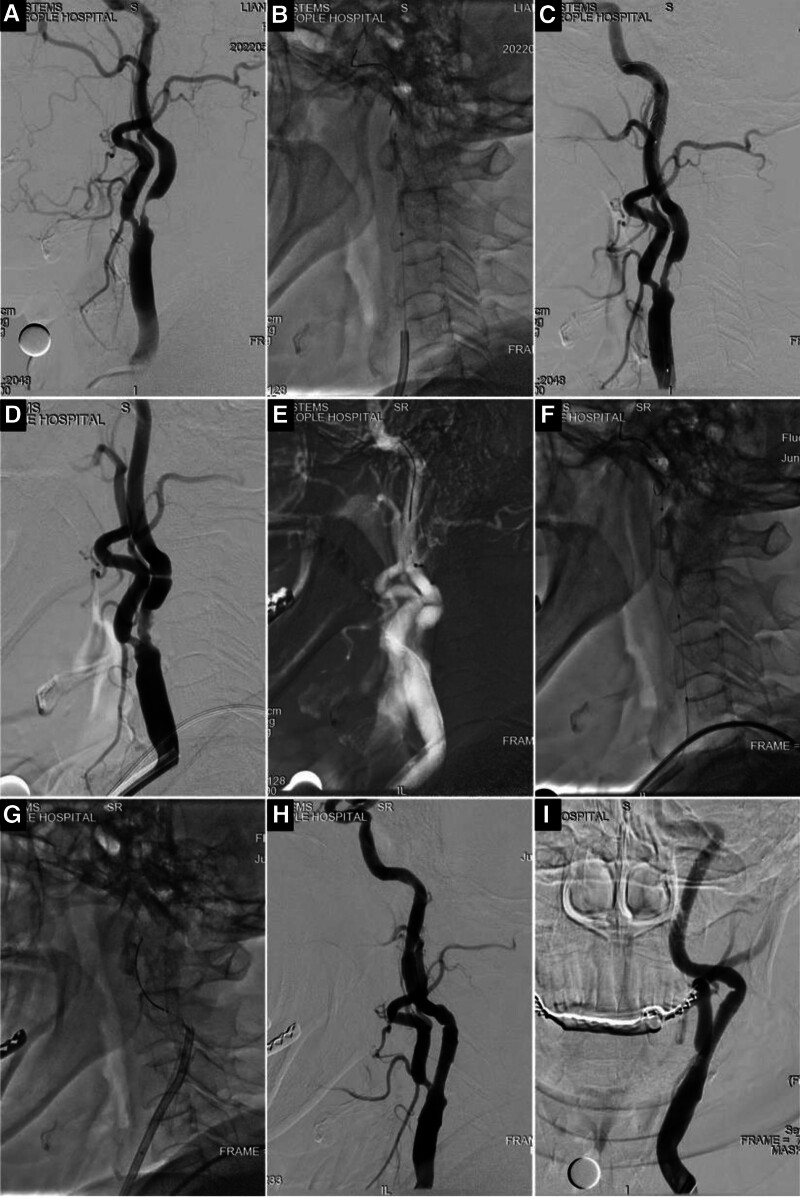
A 66-year-old female. Dizziness for 3 months, aggravated for 1 week. DSA showed severe stenosis at the origin of the left internal carotid artery (A). Place the Emboshield NAV6 embolic protection system (EPS) (B). Left internal carotid artery was dilated by balloon (C). CAS was performed, the initial carotid stenosis was found to be more curved than before (D, triangle) and the contrast staining of the stenosis was uneven (D, arrow). The guide wire and protection system have difficulty and increased tension through tortuous segment (E–G). CAS was performed using the Xact carotid stent system (H and I). CAS = carotid artery stenting, DSA = digital subtraction angiography.

The second case involved a 66-year-old female patient who was admitted on May 22, 2022. due to a complaint of “dizziness for 3 months, exacerbated over the past week.” The symptoms had initially developed 3 months prior, and were described as a constant feeling of lightheadedness that became more pronounced following physical activity. The patient’s symptoms ultimately persisted, prompting her to seek medical attention at a local hospital. MRI of the head showed reduced perfusion in the left cerebral hemisphere and multiple intracranial lacunar infarcts, whereas susceptibility-weighted imaging did not reveal any signs of bleeding. Neck ultrasonography indicated severe stenosis of the left internal carotid artery. On May 25th, cerebral artery angiography revealed severe stenosis at the origin of the left internal carotid artery (Fig. 2A). The distal vessel was 5.7 mm in diameter, and the narrowest point was 0.7 mm, indicating 88% stenosis. Distal blood flow exhibited mild deceleration. SAP was scheduled and commenced with balloon dilation of the left internal carotid artery. An Emboshield NAV6 embolic protection system (EPS) (Abbott, Abbott Park, IL) was initially inserted to provide access to the guidewire and protection system (Fig. 2B). Subsequently, balloon dilation was performed using 2 × 20 mm and 4 × 20 mm balloons, achieving approximately 60% residual stenosis in the left internal carotid artery (Fig. 2C). The intracranial blood flow rate returned to near-normal levels, and the protection system was successfully removed. CAS was performed on June 8th. Intraoperative imaging revealed that the initial stenosis at the origin of the carotid artery was more tortuous than that observed on May 25th (Fig. 2D, triangle). Uneven staining with the contrast agent was observed at the stenotic site (Fig. 2D, arrow). An Emboshield NAV6 EPS (Abbott, Abbott Park, IL) was also utilized, but the guidewire encountered difficulty passing through the tortuous segment. As the guidewire advanced through the tortuous segment, the movement of the protection system along the guidewire became challenging, and increased tension was noted (Fig. 2E). Following implantation, the guidewire for the protection system exhibited a significantly more tortuous path than that previously documented (Fig. 2F). A 5 × 30 mm balloon was used for predilation, followed by CAS using the 9 to 7 × 30 mm Xact carotid stent system (Abbott, Abbott Park, IL). When withdrawing the protection system, the retrieval sheath encountered difficulty in passing through the tortuous segment of the carotid artery. Hence, an 8F guiding catheter was used to navigate via the stent to retrieve he protection system (Fig. 2G). Subsequent angiography revealed resolution of the stenosis in the internal carotid artery (Fig. 2H, arrow). However, the distal end of the stent exhibited a more pronounced tortuosity than that observed prior to balloon dilation (Fig. 2I, triangle). During the perioperative period, the patient’s dizziness was relieved without delirium, irritability, and cognitive function decline. The patient recovered well, and was discharged. During the 3-month follow-up period, the patient did not experience dizziness or any other discomfort. Ultrasound examination of the neck vessels revealed normal blood flow velocity and no stenosis or obstruction.

## 3. Discussion

Hyperperfusion syndrome is one of the most severe CAS complications. In 2009, Japanese scholars reported for the first time that SAP could decrease the incidence of post-CAS hyperperfusion syndrome in high-risk patients with severe carotid artery stenosis.^[8]^ Evidence indicates that a staged treatment approach for severe carotid artery stenosis, involving the CAS 1 to 2 weeks following balloon dilation in the first phase, could enhance cerebral vascular reactivity, consequently lowering the risk of post-CAS hyperperfusion syndrome.^[9]^ Indeed, 1 randomized clinical trial involving 11 domestic centers compared SAP with conventional CAS in high-risk patients with carotid artery stenosis and elevated perfusion. This study included 64 patients, including 31 in the conventional CAS group, and 33 in the SAP group. The incidences of hyperperfusion syndrome in the 2 groups was 9.7% and 0%, respectively. Despite a trend towards reduction, the difference between the 2 groups was not statistically significant. The researchers attributed this lack of significance to the modest sample size, as the study initially aimed to enroll 158 patients, but was prematurely terminated due to enrollment difficulties.^[7]^ Indeed, one meta-analysis involving 1030 patients demonstrated that SAP had a significant advantage over conventional CAS in preventing hyperperfusion syndrome (OR 0.35, *P* = .02), although the difference in distal embolic events between the 2 groups was not statistically significant.^[10]^

In one cohort study of 1004 patients with severe carotid artery stenosis, examinations were conducted to identify individuals exhibiting slow distal flow and impaired collateral flow through digital subtraction angiography, or showing severe reductions in cerebral vascular reactivity and/or cerebral blood flow via perfusion imaging studies using computed tomography, MRI, and/or single-photon emission computed tomography. Eventually, 53 patients were assigned to the SAP group, in which only 2 required immediate CAS due to arterial dissection during balloon dilation, and no other adverse reactions were reported.^[11]^ Of the 535 patients (543 vessels) who underwent single-photon emission computed tomography screening, 113 were assigned to the SAP group and 419 to the CAS group. The incidence of hyperperfusion syndrome was found to be significantly lower in the SAP group than in the CAS group (4.4% vs 10.5%, *P* = .047).

SAP is a relatively straightforward and effective treatment option to prevent hyperperfusion syndrome. In the first case of this study, the patient presented with severe stenosis, while color Doppler ultrasonography revealed a hypoechoic plaque at the origin of the internal carotid artery. The infarction site on head MRI indicated a multifactorial etiology involving both low perfusion and artery-to-artery embolism (cortical infarction). During the first phase of balloon dilation, balloon inflation was smooth, and no dissection was observed throughout the inflation process, indicating a soft texture of the plaque in the internal carotid artery. However, after 2 balloon dilations, there was no apparent improvement in the stenosis. Subsequently, the patient’s symptoms worsened progressively, possibly due to continuous thrombus formation following the rupture of the fibrous cap at the stenotic site after balloon dilation. Due to symptom worsening, the patient underwent CAS 2 days after initial balloon dilation. Despite the absence of predilation, stent deployment was satisfactory, indicating plaque pliability at the stenotic site. Hence, staged treatment may not be suitable for patients with color Doppler ultrasonography findings indicating a hypoechoic plaque in the internal carotid artery and an incomplete fibrous cap. Closed-loop stents may be more appropriate in such cases.

In the second case, during the second-stage CAS performed 13 days after the first-phase balloon dilation, the distal vessels became significantly more tortuous than in the previous examination. This alteration may be attributed to the hemodynamic changes following balloon dilation. In this patient, the Emboshield NAV6 EPS by Abbott was used in both the first and second stages. The deployment and retrieval of the protection system pose challenges, particularly during the retrieval process. The retrieval sheath of the Emboshield NAV6 EPS is relatively rigid, making it challenging to navigate through the tortuous segment. Studies have indicated that patients with a tortuosity index > 80° may encounter difficulties in retrieving the protection system.^[12]^ In the second procedure, the tortuosity of the internal carotid artery exceeded 90° (Fig. 2I), primarily contributing to difficulty in sheath passage. Based on the surgeon’s experience, the retrieval sheath of the Spider FX (Medtronic) or FilterWire EZ (Boston Scientific, Marlborough, MA) is relatively flexible, and appears to be more suitable for these cases. In terms of the protection system design, careful consideration is required to design the retrieval sheath to ensure adequate distal protection. For surgeons responsible for the staged treatment of severe carotid artery stenosis, it is also essential to consider the impact of hemodynamics on blood vessels and select appropriate instruments based on the vascular morphology.

The above 2 patients are both elderly patients, and they are also the first choice for SAP in our center, mainly for the following reasons:

① Physiological changes and risks.

With age, the vascular system of the human body, like other organs, will also undergo a series of physiological changes, such as reduced vascular elasticity, increased atherosclerosis and reduced vascular compliance. For patients with long-term severe carotid artery stenosis, the cerebral blood flow will be significantly reduced, and they will face a higher risk when receiving invasive treatment such as interventional surgery, especially direct CAS, and the occurrence of hyperperfusion syndrome after surgery can directly endanger life. Therefore, for older patients, clinicians may prefer to opt for more conservative treatments such as SAP to reduce the risk of a single treatment.

② Assessment and adaptation.

Elderly patients are often accompanied by a variety of chronic diseases, such as hypertension, diabetes, heart disease, and other diseases. If SAP is performed, the clinician has sufficient time to evaluate and prepare before each intervention to ensure that the patient can tolerate and adapt to the course of treatment. This gradual, phased treatment helps surgeons better grasp the changes in the patient’s condition and adjust the treatment plan in time.

③ Evaluation of efficacy.

For complex vascular diseases, such as severe carotid stenosis with intracranial slow flow or multivessel disease, SAP can provide patients with more accurate and effective treatment. By SAP, the cerebral blood flow of patients can be gradually adapted to the process, improve the treatment effect, and reduce unnecessary complications.

It is important to note that while advanced age is an important consideration in choosing SAP, it is not the only factor. In the selection of treatment, we will also consider the patient’s medical history, physical condition, degree of stenosis and interventional surgery risk, and other factors. For patients with severe intracranial hypoperfusion and intracranial slow blood flow, we will be more cautious.

## 4. Limitations

The present study has some limitations. Firstly, our study adopted a retrospective single-center design with a small sample size and case selection bias, which may weaken the representation of the population in this study. Secondly, this study did not include case–controls, which affected the reliability of the study results. Given these limitations, a multicenter prospective cohort study was conducted in this region. Finally, considering the overall hospital cost of the patient, cerebral perfusion examination was not routinely performed after CAS. Therefore, it is unclear whether the patient has hyperperfusion status.

## Acknowledgments

We thank to the staffs from the Neurology ward of Jiangxi Provincial People’s Hospital for supporting this publication.

## Author contributions

**Data curation:** Yong-Liang Zhou.

**Investigation:** Shi-Min Liu, Xian-Min Cao.

**Methodology:** An Wen.

**Resources:** Wen-Feng Cao.

**Software:** Ling-Feng Wu.
